# Impact of Climate Change on Children’s Health in Limpopo Province, South Africa

**DOI:** 10.3390/ijerph9030831

**Published:** 2012-03-08

**Authors:** Adeboyejo Aina Thompson, Lirvhuwani Matamale, Shonisani Danisa Kharidza

**Affiliations:** Department of Urban and Regional Planning, University of Venda, Private Bag X5050, Thohoyandou, 0950, Limpopo, South Africa; Email: matamelaeth@webmail.co.za (L.M.); dkharidza@webmail.co.za (S.D.K.)

**Keywords:** climate change, children’s health, Limpopo province, South Africa

## Abstract

This paper examines the impact of climate change on children’s health, in the Limpopo Province of South Africa. Twenty one years climatic data were collected to analyse climatic conditions in the province. The study also employs 12 years hospital records of clinically diagnosed climate-related ailments among children under 13 years to examine the incidence, spatio-temporal, age and sex variations of the diseases. Regression analysis was employed to examine the relationships between climatic parameters and incidence of diseases and also to predict distribution of disease by 2050. The results show that the most prevalent diseases were diarrhea (42.4%), followed by respiratory infection (31.3%), asthma (6.6%) and malaria (6.5%). The incidence varied within city, with the high density areas recording the highest proportion (76.7%), followed by the medium (9.4%) and low (2.5%) density residential areas. The most tropical location, Mussina, had the highest incidence of the most prevalent disease, diarrhea, with 59.4%. Mortality rate was higher for males (54.2%). Analysis of 21 years of climatic data show that maximum temperature is positively correlated with years in four cities with r coefficients of 0.50; 0.56, 0.48 and 0.02, thereby indicating local warming. Similarly rainfall decreased over time in all the cities, with r ranging from −0.02 for Bela Bela to r = 0.18 for Makhado. Results of the regression analysis show that 37.9% of disease incidence is accounted for by the combined influence of temperature and rainfall.

## 1. Introduction

The health status of a society is undoubtedly a function of many determinant and influential factors, including biological or genetic composition and socio-economic conditions. However, several studies have shown that linkages exist between climate change and infectious diseases [1,2,3]. For instance, incidence of food borne gastroenteritis and respiratory illnesses are said to increase with increasing temperature [4,5,6]. Also, higher temperature will not only elongate the transmission cycles of some vectors of human diseases [7], and alter the living conditions for animal and plant vectors of diseases [8,9,10], but it will also increase ground level ozone, hasten the onset of pollen season and thus contribute to asthma attacks [11]. Similarly, decrease in rainfall will not only lead to competition among water users [12], but it will affect water quality and thereby increase the risk of water-borne pathogens. Too much rainfall on the other hand will increase incidence of water-borne diseases [13]. It has been concluded that, many of the major killer diseases such as cholera, diarrhea, malaria, dengue and other infections carried by vectors are highly climate sensitive [11], and that climate change may indeed swell the population at risk of malaria in Africa by 90 million by 2030 [14].

While climate change poses serious threats to human societies generally, cities are likely to be more affected than rural areas because of urban air pollution and the greater possibility of heat waves from ubiquitous man-made urban heat islands in the cities [15,16]. The bulk of the burden of climate sensitive diseases are borne by infants and children, as well as the aged and the poor in urban areas [14,17,18,19,20], mostly in developing countries, where low levels of economic development and poor health service coverage compound the problem [14]. The need to focus research attention on the health impacts of climate change on children and the elderly has been stressed [21,22]. According to UNICEF [22], “throughout the world, children face significant threat to health from an array of environmental hazards. The protection of human, *particularly, children who are the most vulnerable* (emphasis ours) remains a fundamental objective of environmental policies to achieve sustainable development” Being the most vulnerable to vagaries of weather, the most exposed and the group with the least resistant ability, the risks children face are likely to be intensified by climate change. The health implication of this becomes more worrisome if we note that in any geopolitical entity, particularly in developing countries or its sub-regions, children constitute a very significant proportion of the population. While 11.3% or 5.4million of the South Africa’s population is found in the Limpopo province, about 2 million or 37.1% of this figure are children under the age of 14 years [23].Further, it is observed that about 12.1% of the provincial populations are children below 5 years. This figure is higher than the estimated national figure for this group in developing countries. According to UNICEF [22], the proportion of children (under five years) that are highly vulnerable (to burden of climate sensitive diseases) range between 10 and 20 percent of the population in countries (or areas of nations) with the least ability to cope with the health implications of climate change. 

It has been observed that 34% of all childhood illness in the World and 36% of deaths in children under age 14 are due to modifiable environmental factors and that because of their physical, physiological and cognitive immaturity, children are more sensitive than adults to harm from environmental hazards [24]. Although, overall death rates for young children continue to drop in most parts of the World due to improved nutrition, health care and immunization rates, as well as better environmental health, for many of the children in the developing countries who are most at risk from diarrhea diseases, respiratory illness, malaria, meningitis, cough, typhoid, measles, and malnutrition (the most significant causes of mortality for children), the situation is likely to worsen with some of the effects of climate change [22]. The point is, climate change increases health hazards by worsening air quality, stimulating more extreme weather events, creating conditions that favour increases in food-water-and vector-borne infections, and enhancing heat stress condition.

While most studies focus on the relationship between climate change and malaria distribution [25,26,27] and analysis of socio-economic costs of malaria [28,29] and cholera [5], the major purview of this study is the examination of the whole spectrum of climate related diseases which affect children. The paper therefore examines the impact of climate change on children’s health, using data from five Municipalities in the Limpopo Province of South Africa. It examines the incidence, prevalence rates, spatiotemporal pattern as well as the relationship between the diseases and climatic parameters of temperature and rainfall. The objectives are to examine the incidence, prevalence rates and trends of the diseases under consideration; examine the intra-urban variation in incidence and prevalence of the diseases; assess the variations in age and sex of the children with respect to prevalence of diseases and mortality rates; examine the relationship between variations in climatic parameters and the diseases; and finally, to suggest adaptation/mitigation strategies.

## 2. The Study Area

The study area of this research is the Limpopo province, the most northerly part of South Africa, with unique geographical location as it incorporates both tropical and subtropical climatic characteristics. The province lies in the great curve of the Limpopo river and is characterized by mixed grassland and trees known as bushveld, majestic mountains, gallery forests and patchwork of farmland. The study covers five out of the nine Municipalities in the province. 

**Figure 1:**
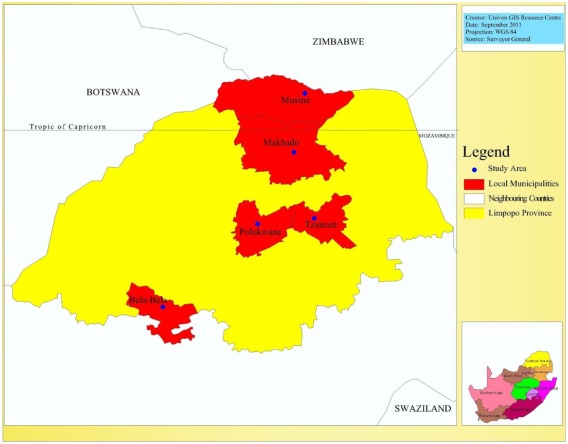
The study area.

As shown in Figure 1, while two of the selected Municipalities, namely the Makhado and Musina local municipalities fall within the tropical region at the northern part of Limpopo, two others, the Capricorn District municipality with headquarters at Polokwane and the Greater Tzaneen Municipality fall around the tropic of Capricorn. Bela-bela City, the headquarters of its local municipality, is the most southerly city of the province, lying within the subtropical region. The District Municipality Hospital in each city was selected for study. These are government hospitals, with comprehensive and relatively longer history of health care programmes, as well as better culture and methods of record keeping.

Each city has geographical and socio-economic peculiarities with possible implications for the prevalence of climate change sensitive diseases and adaptive or coping mechanisms of the people. For instance, Polokwane is not only the largest metropolitan complex, but also the undisputed commercial and industrial enclave in the province. Makhado lies at the foot of the Soutpansberg mountains in a generally fertile agricultural region which produces many varieties of fruits. In the most tropical city in the province, Mussina, mining, tourism and hunting play a vital role in the city’s urban economy. Located within mountain slopes which are heavily forested with pine and blue gum plantations is Tzaneen, the second largest city in the province and the richest sub-tropical fruit-farming region that produces tea, forestry products and tropical fruits [30].

Although a review of the socio-economic differences among the municipalities would have been ideal, given the possible influence of these differences on incidence and prevalence of climate sensitive diseases, however, this is not possible in this paper, due to data constraints. Nevertheless, in each of the five cities, which are the headquarters of their respective municipalities, there are distinct and distinguishable residential neighbourhoods; the urban high, medium and low density residential areas, with well-defined socio-economic and environmental characteristics. Typical of such areas particularly in developing countries, the high density residential area is characterized by planlessness, dilapidated housing conditions, high occupancy ratio and is mainly occupied by the low socio-economic class [31,32,33]. The associated poor living and environmental conditions will promote the transmission of infectious diseases, while the very low *per capita* income will impact negatively on the adaptability and coping capability of the people to climate change related diseases. On the other hand the low density area is characterized by a high socio-economic class living in a well-planned and luxurious home environment [31,32,33]. Both the in-house and immediate environment are equipped and modified to protect children and the aged from vagaries of weather. The medium density residential neighbourhood approximates either the high or low density residential area, depending on stage of city development or prevailing level of socio-economic development.

From the perspective of urban planning therefore, this framework is important for urban policy formulation and programme intervention on climate change related diseases that affect objects of planning efforts, in this case, the children. 

## 3. Research Methodology

Two types of secondary data were required for this study. The first on climate related diseases and the second on climatic parameters. The first data type relates to the incidence of such diseases as malaria, typhoid, asthma, skin cancer, diarrhea, measles, and meningitis. The children covered are those aged 0 to 13 years. Although the study aimed to cover 20 years hospital record from 1990 to 2010, however, the earliest period for which data could be obtained was for 11 years period spanning 1999 to 2010. Our observation is that there is a relationship between age of record and data accuracy and completeness. Record keeping in retrievable format is a recent development (in most cases, not more than 10 years old). In most of the hospitals, older records either do not exist, or had been destroyed or dumped as a heap of documents in inaccessible manner in a “dungeon”. The information gathered include, age, sex, location of patient as shown by residential address, as well as the clinically diagnosed diseases with associated complications. These data types were collected from the medical records section of each of the five hospitals.

The method of collection in each hospital involved a review of case notes of clinically diagnosed diseases. Bearing in mind the age of patient and disease types that were the concern of this study, relevant cases were identified and recorded in a structured questionnaire. A total of 7,869 relevant cases were encountered in the five hospitals during the period covered by the research. 

The second type of secondary data is the 21 years meteorological data covering the period 1990 to 2010. This relates to mean, maximum and minimum temperatures and mean annual rainfall. The climatic parameters were supplied by the South African Weather Services, Pretoria. 

The data collected was subjected to cross tabulation so as to examine the variations in critical variables of interest, while Chi-Square test was specified to test the significance of relationships. Pearson correlation was used to test the relationship between incidence, years of temperature and rainfalls. In order to examine the relationship between climatic parameters and climate related diseases, the incidence of each of the five most prevalent diseases, diarrhea, respiratory Infection, asthma, malaria and meningitis, between 1999 and 2010 was subjected to multiple regression analysis. As a prelude, the data on incidence of diseases was first factor analysed, using the principal component analysis method. This was done to first extract the communalities of the diseases, their variances (measurement of relative importance or prevalence rate) of each disease in the area and then, the derivation of a linear composite factor or component factor—“disease factor”, which is then used as a dependent variable Y1, as an input into a general linear regression model expressed symbolically as Y_1_ = b_1_x_1_ + b_2_x_2_, with x_1_, x_2_ being average temperature and average rainfall respectively are independent variables. The relationship between incidence of disease and climatic parameters of rainfall and temperature is thus examined to test the hypothesized relationship that there is a linear relationship between temperature and rainfall on the one hand and, incidence of diarrhea, respiratory infection, asthma, malaria and meningitis.

In order to predict the distributions of the incidence of diseases, two scenarios were derived; the distribution of incidence with and without influence of climatic parameters. In the latter case, based on observed distribution of diseases incidence between 1999 and 2010, the trend of incidence was projected to 2050. For the second scenario, the trends of observed 21 years rainfall and temperature data (independent variables) were first projected to 2050 before being entered into regression model with each of the observed distribution of diseases as dependent variables.

## 4. Results and Discussion

### 4.1. Trend of Climatic Parameters

**Figure 2:**
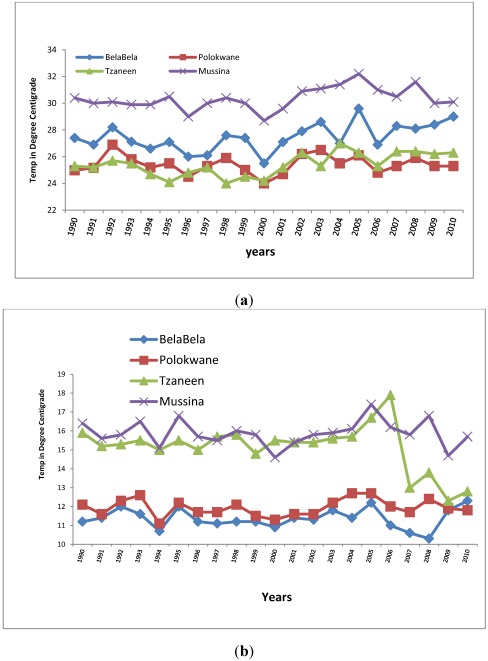
(**a**) Trend in maximum temperature 1990 to 2010; (**b**) Trend in minimum temperature 1990 to 2010.

The trend of climatic parameters in the province was analysed using 21 years of data on temperature and rainfall. As shown in Figure 2a, while expectedly there are fluctuations in maximum temperature during the period, the trend shows an overall increase for the four municipalities for which the data type was available. Temperature data was not available for Makhado Municipality. The R coefficient of variations for each of the cities are 0.50, 0.56, 0.48, and 0.02 respectively for Bela-Bela, Tzaneen, Mussina and Polokwane. The corresponding R coefficients for minimum temperature are 0.004, −0.383, −0.004 and 0.135. The above shows that, while maximum temperature tends to increase during the 21 years period, minimum temperature shows a decrease over the same period, thereby indicating tendency towards local warming which is a possible consequence of climate change in the province. Maximum temperature in the most tropical location, Mussina, was the highest, ranging from 29 °C to 32.2 °C, giving an annual increase of 0.15 °C while for BelaBela, the most subtropical of the locations, maximum temperature ranged from 25.5 °C to 29.6 °C, also giving an annual increase of 0.19 °C. This finding is in line with the IPCC [34] observation to the effect that temperatures have indicated a greater warming trend in Africa since 1960. Also decadal warming rates of between 0.1 °C to 0.3 °C have been observed in South Africa [35]. 

**Figure 3:**
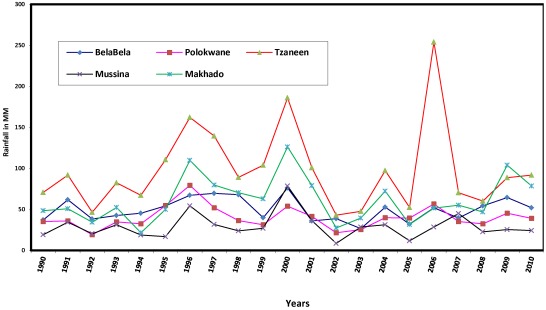
Rainfall trends 1990 to 2010.

Figure 3 shows that although the average annual rainfall fluctuates, it is generally low in the various municipalities during the 21 year period. Tzaneen, a subtropical location, had the highest values, ranging from 43 mm in 2002 to 254 mm in 2006. The relationship between annual rainfall and years was analysed using Pearson correlation. The results show positive but very low coefficients of 0.027, 0.063, 0.020 and 0.188, respectively, for Polokwane, Tzaneen, Mussina and Makhado, and a negative coefficient for BelaBela, the most subtropical location, with R = −0.022. Decreasing rainfall implies that the province is at risk of water stress, which Boko *et al.* [36] noted is being faced by some countries in Africa. The province therefore will add to the 25% of Africa’s population (about 2 million people) that is currently experiencing high water stress, featuring pressure on water availability, water accessibility and water demand. The brunt of this impact would be borne by the children and the elderly, the most vulnerable groups in society.

It is concluded that with increasing maximum temperature, decreasing minimum temperature and low and decreasing average annual rainfall, there are evidences of local warming, a possible consequence of climate change in the province. Observed local warming in the province may be due to land use changes, particularly increasing deforestation again, owing largely to large scale farming, which has greatly altered the very fragile ecosystem of the province. Limpopo province has been described as the garden of South Africa if not of the whole continent, given its rich fruit and vegetable production. The province produces most the country’s mangoes, papaya, tea, avocado, tomatoes and potatoes. Other products such as coffee, nuts, sisal, cotton and tobacco are widely grown. Aside, most of the higher lying areas are devoted to cattle and game ranching [37]. The health implication of climate change featuring local warming and the specific relationships between climatic parameters and incidence of climate change related diseases are some of the issues investigated in the following sections. 

### 4.2. Incidence and Prevalence Rates of Diseases

The data presented in Table 1 and Table 2 show that ten different types of climate sensitive diseases were encountered in the study area. The group of diseases classified as ‘others’ in the tables refer to cases where more than one or two diseases were observed for the same person. For example, a child with complicated cases of diarrhea and malaria or asthma. 

**Figure 4:**
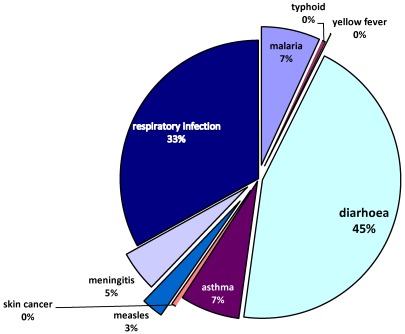
Types of diseases.

As shown in Figure 4, the most prevalent diseases in the study area are diarrhea (42.4%), respiratory infection (31.3%), asthma (6.6%), malaria (6.5%) and meningitis (4.5%). There is also a significant presence of measles (2.4%). The findings here confirm the prevalence of the most important leading causes of death among children (diarrhea, respiratory infection, asthma and malaria) as identified by UNICEF [22]. There are observed links between climatic variations and incidence of such diseases as diarrhea, respiratory infection, asthma and meningitis [22,38,39]. South Africa generally may be a low risk zone for malaria and a medium risk zone for meningitis as mapped by Boko et al. [36], but the Limpopo province in the northern tip exhibits dryness and dusty conditions which predispose populations to the observed infectious diseases. Also, as noted by Mutangadura *et al.* [40], South Africa is still one of the countries in Africa, where a significant proportion of the population still relies on unimproved water sources, a major contributory factor to a range of health problems, including diarrhea. Much of the problem of inaccessibility to safe drinking water and sanitation is borne by the poor women and children living in squalid high density residential areas. 

Further result shows that 99.4% of all incidence of disease affected the black race, while the Coloured, Indians and White race had 0.3%, 0.2% and 0.1% respectively. This variation however reflects more of the general pattern of population distribution in the province, as noted earlier in this report. 

**Table 1:** City variations in incidence of disease.

	NR	Malaria	Dengue	Typhoid	Y. fever	Diarrhea	Asthma	S. cancer	Measles	M‘Gitis	Resinfectn	Others	Total
Mussina No	56	140	7	0	0	863	78	0	115	16	175	4	1,454
R%	3.9	9.6	0.5	0.0	0.0	59.4	5.4	0.0	7.9	1.1	12.0	0.3	18.7
C%	(51.4)	(27.6)	(38.9)	(0.0)	(0.0)	(26.1)	(15.3)	(0.0)	(60.8)	(4.6)	(7.2)	(1.3)	
Makhado No	4	183	1	29	0	229	147	0	5	77	247	40	962
R%	0.4	19.0	0.1	3.0	0.0	23.8	15.3	0.0	0.5	8.0	25.7	4.2	12.4
C%	(3.7)	(36.0)	(5.6)	(85.3)	(0.0)	(6.9)	(28.8)	(0.0)	(2.6)	(22.2)	(10.1)	(12.7)	
Polokwane No	6	5	1	0	0	205	30	0	7	89	29	100	472
R%	1.3	1.1	0.2	0.0	0.0	43.4	6.4	0.0	1.5	18.9	6.1	21.2	6.1
C%	(5.5)	(1.0)	(5.6)	(0.0)	(0.0)	(6.2)	(5.9)	(0.0)	(3.7)	(25.6)	(1.2)	(31.6)	
Tzaneen No	21	102	4	5	1	1598	164	0	8	94	1,918	9	3,924
R%	0.5	2.6	0.1	0.1	0	40.7	4.2	0.0	0.2	2.4	48.9	0.2	50.4
C%	(19.3)	(20.1	(22.2)	(5.6)	(10.0)	(48.4)	(32.2)	(0.0)	(4.2)	(27.1)	78.8	(2.8)	
Belabela No	22	78	5	0	9	407	91	1	54	71	66	163	967
R%	2.3	8.1	0.5	0.0	0.9	42.1	9.4	0.1	5.6	7.3	6.8	16.9	12.4
C%	(20.2)	(15.4)	(27.8)	(0.0)	(0.1)	(12.3)	(17.8)	(100)	(28.6)	20.5	(2.7)	(51.6)	
TOT	109	508	18	34	10	3295	510	1	189	347	2,435	316	7,780
	1.4	6.5	0.2	0.4	0.1	(42.4)	6.6	0.0	2.4	4.5	31.3	4.1	100

X^2^ = 0.000.

**Table 2:** Incidence of disease by residential density.

	NR	Malaria	Dengue	Typhoid	Y. fever	Diarrhea	Asthma	S. cancer	Measles	M’ Gitis	Resinfectn	Others	Total
NR No	9	196	2	26	2	214	163	0	21	78	94	83	888
R%	1.0	22.1	0.2	2.9	0.2	24.1	18.4	0.0	2.4	8.8	10.6	9.3	11.4
C%	(8.3)	(38.6)	(11.1)	(76.5)	(20.0)	(6.5)	(32.0)	(0.0)	(11.1)	(22.5)	(3.9)	(26.3)	
LOW No	7	15	0	0	0	115	11	0	1	12	18	13	192
R%	3.6	7.8	0.0	0.0	0.0	59.9	5.7	0.0	0.5	6.3	9.4	6.8	2.5
C%	(6.4)	(3.0)	(0.0)	(0.0)	(0.0)	(3.5)	(2.2)	(0.0)	(0.5)	(3.5)	(0.7)	(4.1)	
MED No	23	39	6	0	3	341	44	0	7	43	192	32	730
R%	3.2	5.3	0.8	0.0	0.4	46.7	6.0	0.0	1.0	5.9	26.3	4.4	9.4
C%	(21.1)	(7.7)	(33.3)	(0.0)	(30.0)	(10.3)	(8.6)	(0.0)	(3.7)	(12.4)	(7.9)	(10.1)	
HIGH No	70	258	10	8	5	2,625	292	1	160	213	2,129	188	5,959
R%	1.2	4.3	0.2	0.1	0.1	44.1	4.9	0.0	2.7	3.6	35.7	3.2	76.7
C%	(64.2)	(50.8)	(55.6)	(23.5)	(50)	(79.7)	(57.3)	(0.0)	(84.7)	(61.6)	(87.5)	(59.5)	
TOT	109	508	18	34	10	3,295	510	1	189	346	2,433	316	7,769
	1.4	6.5	0.2	0.4	0.1	42.4	6.6	0.0	2.4	4.5	31.3	4.1	100

X^2^ = 0.000.

As shown in Table 1, the profile of the municipalities on disease incidence varies. For instance, more than half (59.4%) of disease incidence in the most tropical location, Mussina, was diarrhea, which was the most prevalence disease in the province. The proportion was over fourty percent in Polokwane (43.7%), BelaBela (42.1%) and Tzaneen (40.7%), which also had the highest concentration of the second most prevalent disease, respiratory infection, with 48.9 per cent, followed by Makhado (25.7%) and Mussina (12%). The observed intercity variations in disease incidence is statistically significant, with a *p*-value of 0.00.

**Figure 5:**
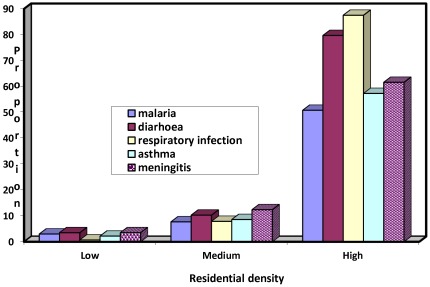
Intracity variations in incidence of most prevalent diseases.

Further analysis as summarized in Table 2 reveals that the incidence of disease also varies within residential areas, with the high density neighbourhood recording the overall highest proportion of 76.7 per cent, followed at a distance by the medium and low density areas, respectively, with 9.4 and 2.5 per cent. The observed intracity variation is statistically significant, with a p-value of 0.00. Figure 5 illustrates the profile of the density areas on the five most prevalent diseases. The high density areas had the highest proportion of diarrhea (79.7%), respiratory infection (87.5%), asthma (57.3%) malaria (50.8%). In each of these cases, the medium and low density areas had the lower and least proportion respectively. 

### 4.3. Sex and Age Variations in Incidence of Disease

**Figure 6:**
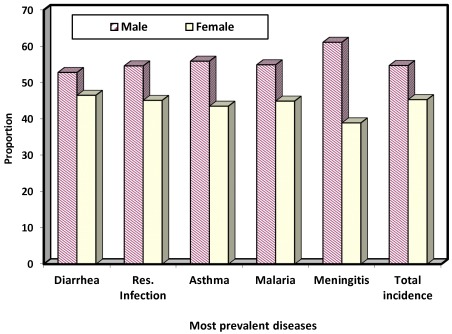
Sex variations in incidence of disease.

Figure 6 shows that more than half of the children who suffered from climate change related disease were males (54.7%). Also on the five most prevalent diseases, male children had higher proportion with 52.8%, 54.6%, 55.9%, 54.9% and 62%, respectively, for diarrhea, respiratory infection and malaria. The observed sex variation in incidence of disease is statistically significant, with a *p*-value of 0.00. This implies that male children are more susceptible to climate change related diseases than their female counterparts. This is perhaps as a result of the fact that male children particularly from ages 3 upward are more active in and interactive with the environments and consequently more exposed to the vagaries of weather. The relationship between ages and disease incidence is investigated next.

**Figure 7:**
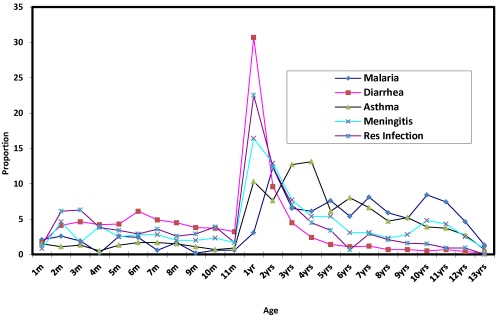
Relationship between age and incidence of disease.

Figure 7 shows the age specific incidence of five most prevalent diseases among the children. It is crystal clear that ages 1 to 2 years were more susceptible than other ages in all the five most prevalent cases. Thus this age group is considered very critical as far as climate change related diseases, particularly diarrhea, respiratory infection, meningitis and malaria. However, the result of Pearson correlation between the ages and incidence of the five disease shows that, malaria, asthma and meningitis respectively with r = 0.537, 0.364 and 0.073 are positively correlated with age. This implies that the older a child is, the more susceptible it is to malaria, asthma and meningitis. This is to be expected since older children spend more time than the younger children, outside the home environment in the street or neighbourhood for play and other outdoor activities and are thus exposed to the vagaries of weather. On the other hand, diarrhea and respiratory infection, each with r = −0.245 and −0.205, have a negative correlation with age. In the case of diarrhea, this is to be expected since older children tend to show greater sense of responsibility in the area of environmental hygiene than the younger ones, while higher incidence of respiratory infection among the younger children may be due to lower immunity against airborne pathogens which causes the infection.

### 4.4. Trend in Incidence of Disease

**Figure 8:**
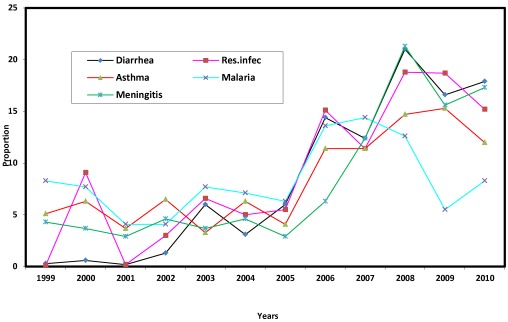
Trend in incidence of most prevalent diseases.

Figure 8 shows the trend in the five most prevalent diseases. That there was increasing incidence of the five most prevalent diseases is indicated by the results of Pearson correlation, which are 0.928, 0.930, 0.813, 0.392 and 0.818, respectively, for diarrhea, respiratory infection, asthma, malaria and meningitis. While all cases were below 10 per cent up till 2005, there was upsurge thereafter, with diarrhea and meningitis reaching epidemic proportions in 2008, before showing a slight decrease in 2009 and a tendency to rise again in 2010. The observed upsurge in incidence may be due to an increase in local temperature, as observed earlier. The apparent ineffectiveness of the existing health care policy is however indicated, signaling a dire consequence on the health of the children. It is these consequences that are investigated next. 

### 4.5. Consequences of Diseases on Children

**Figure 9:**
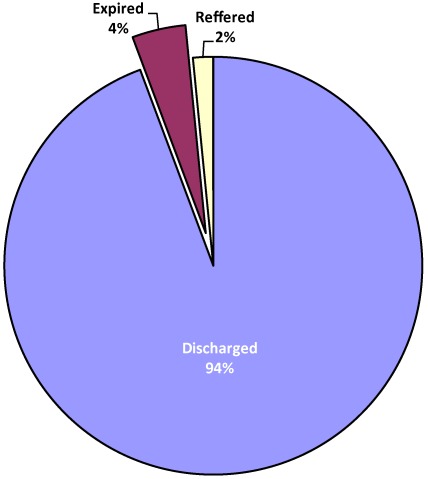
Consequencies of disease.

Result of analysis as illustrated in Figure 9 shows that out of the children admitted for climate change related diseases, 91.3 per cent were successfully treated and discharged, while a significant proportion 4.0% died of the various diseases. Another 1.5 per cent were referred to other hospitals or had to be discharged based on request of parents. Result of further analysis as summarized in Table 3a–c reveals that of those children that died, 54.2% were males while 44.9% were females. (sex of remaining 0.7% not indicated). The observed sex variations in mortality rates is significant with *p*-value of 0.00.

**Table 3a:** Summary of regression model.

R	R square	Adjusted R Square	Std. error of the estimate
0.616 ^a^	0.379	0.241	0.87092738

^a^ Predictors: (constant), Av Rainfall, Max Temp.

**Table 3b:** ANOVA.

Model	Sum of Squares	Df	Mean Squares	F	Sig.
1 Regression Residual Total	4.173 6.827 11.000	2 9 11	2.087 0.759	2.751	0.117 ^a^

^a^ Dependent Variable: REGR factor score 1 for analysis 1.

**Table 3c:** Regression coefficients ^a^.

Model	Unstandardised Coefficients		Standardized Coefficients	T	Sig
	B	Std. Error	Beta		
1 (constant)	−38.681	16.546		−2.338	0.044
Max. Temp	1.329	0.571	1.039	2.329	0.045
Av. rainfall	0.041	0.020	0.914	2.048	0.071

^a^ Dependent Variable: REGR factor score 1 for analysis 1.

In line with earlier observation, 98.1 per cent of the deaths occurred among the black children, while most of the deaths 96.7% resulted from dehydration associated with diarrhea, the most prevalent disease in the study area. The finding here is in line with UNICEF [22], which identified diarrhea and respiratory infection as the most important and leading causes of mortality among children.

### 4.6. Relationship Between Incidence and Climatic Parameters

Result of the factor analysis reveals that there is only one linear composite, which reliably extracts 81.21% variance from the data set. Table 4 shows the composite factor and the loading of the diseases. In the explanation of the relationship between the diseases and climatic parameters, the most important disease as also noted in earlier discussion of prevalent rates of diseases is diarrhea (0.979), followed by respiratory infection (0.963), asthma (0.951), meningitis (0.928) and malaria (0.639). 

**Table 4:** Communalities explained by each disease.

	Component score
Diarrhea	0.979
Respiratory Infection	0.963
Asthma	0.951
Malaria	0.639
Meningitis	0.928

Extraction Method: Principal Component Analysis

The result of regression model as shown in Table 3a reveals that while the coefficient of joint correlation R, between the composite diseases and climatic parameters is 0.616, the coefficient of determination R^2^ is 0.379. This implies that 37.9% of incidence of disease may be attributable to climatic parameters. However, Table 3b shows that with F ratio of 2.751 and p = 0.117, the observed relationship is not statistically significant at 95% confidence level. The point to note here is that, temperature and rainfall are major closely associative factors with incidence of disease. Since about 40 percent of disease incidence is accounted for by climatic parameters, if these vital parameters are controlled, close to half of incidence will be under control.

Thus calibrating the predictive model in Table 3c, y = a + bx + e, where y = the linear composite of the diseases (diarrhea, respiratory infection, asthma, meningitis and malaria in order of importance), y = −38.681 + 1.329 (maxtemp) + 0.41 (avrain) + e.

The result implies that, if all the climatic parameters are kept constant, and all other things being equal, particularly, the current level of development including health care and other infrastructural provision, a unit increase in temperature will produce a 1.329-fold increase (more than 100%) in the incidence of the five major diseases. Given the fact that local temperature is increasing this observation portends great danger for the children in the province. Perhaps it is not out of place to say that the children in the area may be living in a state of emergency as any further increase in temperature, a very certain occurrence would spell further doom on the children’s health. The importance of a healthy children population cannot be overemphasized, being the hope of the nation, the soul and vigour of the subregion.

It is however instructive to note that, since only 40 percent of variations in the disease incidence is accounted for by climatic parameters, the remaining 60 percent variations may be attributed to a combination of socio-economic and environmental factors summarized in poverty as the types of diseases considered here are among those described as poverty related diseases [41,42] and defined as several groups of infectious diseases that put a great burden on developing countries and hamper economic development. These diseases include a large number of tropical diseases such as malaria, diarrhea, typhoid, *etc*. that predominantly affect children in developing countries. The diseases are said to be more prevalent among the poor than among the wealthier people [43], and as such poverty is said to be the leading risk factor as well as consequence of poverty related diseases in developing countries [42,44]. That the bulk of people affected by climate sensitive diseases in the study area are the poor is understood in this context. The point is, for reasons including crowded living and working conditions, inadequate sanitation, particularly in the high density residential areas, the poor are more exposed to climate sensitive and poverty related infectious diseases. While such diseases may result directly from poverty, malnutrition can hinder recovery and exacerbate the diseases, just as the diseases can perpetuate and deepen impoverishment by sapping personal and family resources, thus emphasizing the dynamic relationship between poverty, poor health and climate sensitive infectious diseases [43,44]. However, further research is required in the study area on the relationship between poverty and the related diseases.

### 4.7. Predicted Distribution of Diseases

The future distribution of the five most prevalent diseases are predicted under two scenarios: the first is the trend of distribution without the influence of climatic parameters of rainfall and temperature, and the second scenario takes these influences into consideration

**Figure 10:**
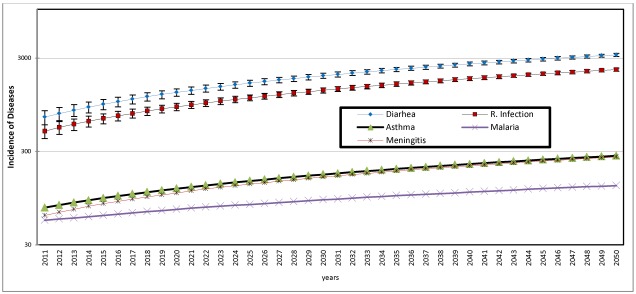
Projected distribution of diseases 2011 to 2050.

**Table 5:** Projected prevalence rates of diarrhea and respiratory infection.

Year	Projected Pop of Children under 5 years	Diarrhea	Respiratory Infection	Projected Diarrhea prevalence rate (%)	Projected Respiratory infection rate (%)
2010	713,551	591	370	0.08	0.05
2015	827,202	961	677	0.1	0.08
2020	958,955	1,288	903	0.13	0.09
2025	1,111,693	1,615	1,129	0.14	0.1
2030	1,288,759	1,942	1,355	0.15	0.1
2035	1,494,026	2,269	1,581	0.15	0.1
2040	1,731,988	2,596	1,807	0.15	0.1
2045	2,007,851	2,923	2,033	0.15	0.1
2050	2,327,653	3,250	2,258	0.14	0.1

The results of the first scenario, as illustrated in Figure 10, are the predicted values based on observed trend of each of the diseases between 1999 and 2010. Since the 12 year trend shows a consistent increase with time, as indicated by positive r coefficient, as observed earlier in Section 4.4, the predicted values are the linear functions of the distribution. Figure 10 reveals the dominating prevalence of diarrhea and respiratory infection and that from 2011 to 2050, these diseases will progressively increase and perhaps reach an epidemic proportion by 2050 if not brought under control. In order to highlight the implications of this observation, the prevalence rates of diarrhea and respiratory infection among the children below 5 years of age is calculated using the 2007 mid-year estimated population of this cohort as provided by SA statistics [23] and using a conservative children population growth rate of 3.0 percent to project the population up to year 2050 and express the disease incidence as a quotient of the population. The results summarized in Table 5 show that from a prevalence rate of 0.08 per cent of population of children below 5 years in 2010, prevalence of diarrhea will increase to 0.1 per cent in 2020 and 0.2 per cent by 2050. The prevalence rates for respiratory infection will increase slightly, from 0.05 per cent in 2010 to 0.1 by 2050. The above implies that by 2050 about one in every one thousand children will suffer from these diseases if the current rate is maintained. However, the prevalence rate will be much higher, if the population of children below 5 years (the most affected group as observed earlier in this paper) is considered. 

**Figure 11:**
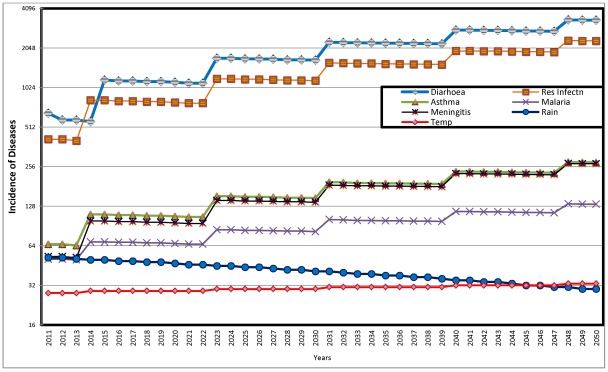
Predicted Distribution of Diseases with Influence of Climatic Parameters 2011 to 2050.

The result of the second scenario, the predicted distribution of diseases taking into consideration the influence of climatic parameters is illustrated in Figure 11. The figure shows that while average annual rainfall decreases over time from 57 mm in 2010 to 41 mm, 35 mm and 30 mm respectively in 2030, 2040 and 2050, the corresponding average temperature figures for the four periods are 28 °C, 30 °C, 32 °C and 33 °C. Again, as observed in the first scenario, the dominating prevalence of diarrhea and respiratory infection is shown in Figure 11. All the diseases show fluctuating but increasing trend between 2011 and 2050. The fluctuations are perhaps in response to extraneous factors, including trend of predicted average temperature and average annual rainfall. The implications of the observations here is that, as temperature increases and rainfall decreases, there are significant increase in the incidence of diarrhea, respiratory infection and slight increase in incidence of asthma, malaria and meningitis. 

For more emphatic conclusions, Table 6 compares the predicted incidence of two most predominant diseases, diarrhea and respiratory infection, with and without climatic influence for selected periods. The major observation from the table is that while the predicted incidence without climatic influence shows a steady increase throughout the period, there are fluctuations in incidence associated with periodic increases in temperature. For instance, while temperature increases from 2 °C in 2011 to 29 °C in 2015, diarrhea also increases by about 79 percent (from 652 to 1,166) over the same period. It will however decrease to 1,122 in 2020, when temperature remains at 29 °C by this period. Respiratory infection shows similar pattern over the same period. Again, with increase in temperature from 31 °C in 2035 to 32 °C in 2040, diarrhea will increase by about 26 per cent and respiratory infection by the same proportion while both diseases show slight decrease when temperature remains at 32 °C up till 2045. 

**Table 6:** Predicted incidence of major diseases with and without climatic influence for selected periods.

Selected periods	Incidence without climate influence *		Incidence with climate influence **		Av rainfall (mm)	Av Temp (°C)
	Diarrhea	Res. Infec	Diarrhea	Res. Infec		
2011	700	496	652	413	57	28
2015	961	677	1,166	819	50	29
2020	1,288	903	1,122	789	47	29
2025	1,615	1,129	1,695	1,185	44	30
2030	1,942	1,355	1,652	1,154	41	30
2035	2,269	1,581	2,224	1,550	38	31
2040	2,596	1,807	2,798	1,946	35	32
2045	2,923	2,033	2,754	1,916	32	32
2050	3,250	2,258	3,342	2,322	30	33

* Predicted values based on observed trend. ** Predicted values based on regression model Source: Authors 2011.

Against earlier observations of warming trend in South Africa [35], a phenomenon which has been noted to improve conditions for malaria transmission and other highland epidemics [5,6], the increasing warming trend predicted here portends great danger for the children in the province.

## 5. Summary Recommendations

### 5.1. Summary

From the analysis and discussion in this paper, the most prevalent climate change related diseases in the study area were diarrhea, respiratory infection, asthma and malaria. The total incidence varied within city, with the high density areas recording the highest proportion, followed by the medium and low residential density areas. Also the most tropical location, Mussina, had the highest incidence of most prevalent disease, diarrhea, followed by the most subtropical location, Bela-Bela. Mortality rate was higher for male than female children. Analysis of 21 years of temperature and rainfall data for the municipalities show that maximum temperature is positively correlated with years, while rainfall decreases overtime, thereby indicating local warming in the Province. Incidence of disease tends to increase with temperature while showing a very low correlation with rainfall. Result of multiple regression shows that 37.9% of incidence of disease may be attributable to climatic parameters. In particular, the most important climatic parameter that greatly influences incidence of disease is temperature, which unit increase would result in over 100 per cent increase in incidence of diarrhea, respiratory infection, asthma, meningitis and malaria, which according to UNICEF [22] are the leading causes of death in children. There is however the need for further research on the varying levels of awareness of health implications of climate change as well as the adaptation and coping strategies of the communities in the province. 

### 5.2. Recommendations for Mitigation/Adaption Strategies

The analysis and discussion in this paper point to the fact that there are climatic variations in Limpopo province featuring, increasing maximum temperature and decreasing rainfall which are suggestive of local warming and possible consequences of climate change. It is also clear that temperature is significantly associated with incidence of diseases. Consequently climate change has significant influence on children’s health.

- Mitigation and adaptation strategies or primary and secondary preventive measures are generally suggested strategies in pediatric health care to address the effects of climate change on children. While mitigation involves reducing Greenhouse Gas (GHG) concentrations in the atmosphere with the goal of reducing climate change, adaptation involves developing public health strategies to minimize and in some cases eliminate local and regional adverse health outcomes that are anticipated from climate change. We suggest both immediate, medium and long term strategies to address climate change related illnesses in children, and that different levels of responses identified here, are to be involved so as to ensure sustainable responses to threats of climate change on children’s health in the province. These levels of responses are: the family circle, the community, local municipality, NGOs and International Agencies.
- Of immediate concern at the primary family circle level is recognition of the fact that parents have the responsibility of ensuring that their children are not exposed to a contaminated environment where their children live or play. It is observed in this study that children under 5 years of age are the most affected compared to the older age groups. There is therefore the need to embark on enlightenment campaign on care for this age group. This should be a consciously formulated programme of action packaged for health care providers and administered through existing health care networks at the community level with special attention on the high density areas. This effort can be complemented by activities of NGOs and other International agencies in different communities in the province.
- Also there is need to improve the children’s level of health within the family circle. This implies improvement in family’s socio-economic status and by logical extension capacity to cope with adverse effects of whether on their children, improvement in health care services particularly those aspects affecting children.
- As shown in this study, the bulk of the people affected by the disease are mainly the poor in the high density residential neighbourhoods. Consequently, there is the need for the formulation of a massive support for sustainable job creation while the existing ones can be made more sustainable. This can be a sustainable pathway for the eradication of poverty, a major cause and consequence of pervasive and persistence poor health. The role of NGOs and International agencies here is of utmost importance.
- Communities on the other hand should be organized through the various traditional leaders/authority and with the assistance of local and district municipalities and NGOs to embark on good environmental practices such as regular environmental sanitation. The Municipalities should provide the necessary legislative and administrative frameworks, while logistic and infrastructural support be provided by the NGOs. In order to inculcate desirable culture of clean in-house and neighbourhood environment, there should be the creation of sanitary inspectorate division or unit at each municipality. The inspectors are to pay regular visits to homes and neighbourhoods to enforce clean environment policies. No doubt, a clean environment would not give room for disease vectors to breed.
- As a medium and long term measure is the need to control pattern of human settlement developments such that developments on mountains, hill sides, alongside the valleys and other areas prone to erosion are avoided. The current pattern of settlement developments in many parts of the province, particularly in such parts of Makhado Municipality such as as Tshakhuma and Tsianda, will obviously accelerate soil erosion and deforestation. Also there is the need to focus policies and programmes on improving the physical environmental conditions of those in the high density areas, where over 90 per cent of incidence are recorded. Perhaps there is the need to declare the high density areas, as disaster risk areas and target them for health improvement plans and actions.
- Closely related to the above is the need for environmental education aimed at minimizing or the prevention of wild veld fires and deforestation. Given the fact that several communities in the Limpopo province still have areas in which people embark on indiscriminate cutting-down of trees as they generally rely on fuel wood for domestic purposes. There is therefore the need to pursue tree planting campaign more vigorously at the grassroots through traditional authorities.
- Of both medium and long term strategies is the need for awareness regarding issues of implications of climate change. Communities, local municipalities and indeed the general public should be aware of the impact that climate change has on their health and on the environment in which they reside. Creation of awareness of climate change can be done if the general public itself is allowed to participate together with government in the discussions of coming up with strategies that can curb the impacts of climate change. Specifically, there is the need to involve women and children, particularly those in the primary school in decision making and programmes aimed at improving children’s health. This will ensure that relevant stakeholders in children’s health, mothers in particular are not only informed but are involved in responding to the challenges that affect their lives.
- A major research direction is the investigation of the varying levels of awareness of climate change issues and household response patterns in the province.

## 6. Conclusions

The observed change in climate in the province calls for attention. The temperature is getting warmer and rainfall is decreasing. Climate change is a reality in the province. This in itself has very wide implications, not only for human health, but also for agriculture as the area is generally known as a major food basket of the nation. With specific reference to health, increasing dryness will increase the incidence of air borne and communicable diseases. Children will be the worst off as they are the most exposed and most vulnerable and the least equipped to cope with the health hazards, given their rather weak socio-economic background. There is therefore the need to focus policy and programme attention on children in the province, particularly those of them in the low class but high density residential areas. 
